# Genome-wide comparative analysis of variability and population structure between autochthonous Turkish chicken breeds and commercial hybrid lines

**DOI:** 10.1016/j.psj.2025.105193

**Published:** 2025-04-19

**Authors:** Eymen Demir, Bahar Argun Karsli, Demir Özdemir, Umit Bilginer, Huriye Doğru, Sarp Kaya, Veli Atmaca, Nimet Tufan, Ebru Demir, Taki Karsli

**Affiliations:** a Department of Animal Science, Faculty of Agriculture, Akdeniz University, Antalya, 07070, Republic of Türkiye; b Department of Agricultural Biotechnology, Faculty of Agriculture, Eskişehir Osmangazi University, Eskişehir, 26160, Republic of Türkiye; c Department of Agricultural Biotechnology, Faculty of Agriculture, Akdeniz University, Antalya, 07070, Republic of Türkiye; d Department of Animal Science, Michigan State University, East Lansing, MI 48824, USA; e Department of Medical Services and Techniques, Vocational School of Burdur Health Services, Burdur Mehmet Akif Ersoy University, Burdur, 15100, Republic of Türkiye; f Department of Animal Science, Faculty of Agriculture, Eskişehir Osmangazi University, Eskişehir, 26160, Republic of Türkiye

**Keywords:** Chicken, ddRADseq, NGS, Genetic diversity, Population divergence

## Abstract

Next-generation sequencing (NGS) technologies have revolutionized livestock genomics by enabling rapid, high-resolution genotyping of local populations with thousands of single nucleotide polymorphisms (SNPs), offering unprecedented accuracy and cost efficiency. This study presents the first comprehensive genomic assessment of the Denizli (DNZ) and Gerze (GRZ) chicken breeds, comparing them to commercial broiler and layer hybrid lines using the double digest restriction-site associated DNA sequencing (ddRADseq) technique. A total of 94,208 bi-allelic SNPs were common between DNZ and GRZ, while 33,284 SNPs were retained among all populations after the quality filtering process. Genetic diversity parameters were higher in native Turkish chicken breeds compared to hybrid lines in which minor allele frequency (MAF) was higher than 0.3 in DNZ and GRZ while it was lower than this value in commercial hybrid lines. Notably, DNZ displayed the highest observed (0.386) and expected (0.375) heterozygosity, whereas the broiler hybrid line showed the lowest heterozygosity (0.254), suggesting inbreeding depression (FIS = 0.241). The negative inbreeding coefficient values occurring due to random mating were observed in DNZ and GRZ chicken breeds, while this value was estimated at 0.118 in the layer hybrid line. Population structure analyses such as principal component analyses (PCA), genetic distance-based neighbor-joining (NJ) tree, ADMIXTURE, and TreeMix algorithm revealed that DNZ and GRZ were genetically distinct from both each other and commercial hybrid lines. The results of this study confirm that comprehensive conservation strategies are efficient approaches to keeping genetic variability at an optimal level without inbreeding. Moreover, this study demonstrates the efficacy of ddRADseq in generating high-throughput genotypic data, providing a cost-effective framework for genomic diversity and population structure studies in indigenous chicken breeds.

## Introduction

Since their domestication, chickens have played a crucial role in performing religious ceremonials and cultural traditions. However, their primary function has shifted toward commercial production, serving as a key source of animal-derived protein through egg and meat production (Karslı et al., 2020). While traditional poultry farming remains integral to rural communities in developing and underdeveloped regions (Padhi, 2016), the global poultry industry is increasingly dominated by intensively selected commercial broiler and layer lines to meet rising consumer demand (Korver, 2023). Being developed via long-term and intensive selection practices, commercial lines have advantages over native chicken breeds, leading to significant decreases in their population size over generations (Franzoni et al., 2021). This kind of instant and continuous reduction in effective population size has brought several local chicken breeds to the danger of extinction (Mahammi et al., 2016). In this regard, comprehensive and well-designed conservation studies have significant potential to eliminate the risk of extinction in local livestock populations by suitable mating programs to increase heterozygosity and decrease inbreeding levels (Demir and Balcioğlu, 2019). However, the success of conservation programs depends on evaluating detailed phenotypic data and genome-wide genetic characterization (Maharani et al., 2021; Ghildiyal et al., 2023).

Rapid advancements in molecular genetics have enabled the assessment of genetic variability and population structure in local livestock breeds using high-density single nucleotide polymorphisms (**SNPs**) derived from SNP arrays and next-generation sequencing (**NGS**) technologies (Bilginer et al., 2022). Among NGS-based approaches, double digest restriction-site associated DNA sequencing (**ddRADseq**) has emerged as a powerful tool, utilizing enzymatic digestion with two restriction enzymes to reduce genome complexity while facilitating high-throughput genotyping of animals with thousands of SNPs in a cost-effective manner (Argun Karslı et al., 2024).

As underlined by Özdemir and Cassandro (2018), several local chicken populations survive across rural areas of Türkiye while only Denizli (**DNZ**) and Gerze (**GRZ**) have been officially categorized as distinctive breeds. DNZ breed is reared in several parts of Anatolia in Denizli province particularly while GRZ husbandry is practiced by villagers of Sinop province and nearby areas of the Black Sea region. Both native chicken breeds are kept by smallholder farmers not only to produce animal-derived products (eggs and meat in particular) but also for ornamental purposes. Indeed, DNZ cocks possess different patterns in terms of feather colors on the hackle, neck, and breast while they are of the ability to perform long crowing activities for nearly 25 s (Özdemir and Cassandro, 2018). It is reported that this kind of long-crowing activity may sometimes lead animals to become unconscious (Karaman and Kirdag, 2012). GRZ chickens possess a whiteness behind their ears, while the beak, tibia, and foot are completely black, with a body covered by black feathers (Onbaşılar et al., 2019). Although native Turkish chicken breeds are well-adapted to the environmental stressors of Anatolia, significant decreases have occurred in their population size. Therefore, DNZ and GRZ breeds were subjected to a national conservation program operated by the Lalahan Livestock Central Research Institute in 1997. As aforementioned, comprehensive conservation programs require detailed phenotypic and genome-wide genotypic characterization in which sufficient scientific studies are available to assess the phenotypic traits of DNZ and GRZ breeds (Özdoğan et al., 2006; Özdoğan et al., 2007; Tuncer et al., 2008; Kaplan and Aksoy, 2009; Garip et al., 2011; Onuk et al., 2017; Arslan et al., 2023). By reviewing the literature, on the other hand, it was detected that the genetic variability in DNZ and GRZ breeds has been evaluated mainly by microsatellite markers (Özdemir et al., 2016; Özdemir and Cassandro, 2018) and mitochondrial DNA (**mtDNA**) (Karaman and Kirdag, 2012; Meydan et al., 2016), whereas no studies have been carried out at the genome-wide level. Therefore, using the ddRADseq technique, this study aims for the first time to investigate the genomic diversity and population structure of DNZ and GRZ chickens at the genome-wide level via comparative analysis with commercial broiler (**BROHIB**) and layer (**LAYHIB**) hybrids. Moreover, this study is the first attempt to use the ddRADseq technique to assess genetic variability and population structure in local chicken breeds and commercial hybrids.

## MATERIAL AND METHODS

### Ethics statement

This study was reviewed and approved by the Local Ethics Committee of Animal Experiments of the Eskişehir Osmangazi University (Grant Number: HADYEK-949/2023)

### Animal sampling and DNA isolation

A total of 120 chickens, representing both sexes, were selected for genomic diversity and population structure analyses: DNZ (*n* = 30), GRZ (*n* = 30), broiler hybrid (BROHIB, *n* = 30), and layer hybrid (LAYHIB, *n* = 30). The DNZ chickens were sampled from independent breeder stocks affiliated with the Denizli Rooster Breeders Association, under the Turkish Hobby and Ornamental Chicken Federation in Denizli province. The GRZ chickens were obtained from Gerze Hacıkadi Company, which maintains a pure stock conservation program for the breed in Sinop province. Both DNZ and GRZ chickens were sampled from six distinct populations, based on breeder interviews to minimize inbreeding. In contrast, BROHIB and LAYHIB chickens were sourced from commercial enterprises located in Eskişehir and Afyon provinces, respectively. Blood samples collected from the wing vein were subjected to the GeneJET Genomic DNA Purification Kit (Thermo K0721) to isolate total DNA.

### Genomic library preparation and sequencing

The standard ddRADseq protocol explained by Peterson et al. (2012) was followed with minor modifications to prepare genomic libraries. In the first stage, the Qubit 4^TM^ fluorometer (ThermoFischer Scientific) with DNA HS assay kit (Invitrogen) was employed to quantify the isolated DNA samples. Subsequently, enzymatic digestion of DNA samples was performed in a 20 µl reaction (17 µl total DNA, 0.5 µl *EcoR*I, 0.5 µl *Msp*I, and 0.5 µl Cutsmart Buffer) incubated overnight at 37°C, while the remnants of restriction enzymes and buffer solution were cleaned by the AmPureXP beads (Beckman Coulter). Twenty-four specific adapters including five base barcodes were ligated to the DNA fragments by using T4 DNA ligase. Adapter ligation was carried out in 40 µl reaction (33 µl DNA fragments, 1 µl molecular barcode, 5 µl 10X ligase buffer, and 1 µl T4 DNA ligase) subjected to an optimized polymerase chain reaction (**PCR**) conditions: initial denaturation at 98°C for 1 min, followed by 17 cycles at 98°C for 20 s, at 65°C for 30 s and at 72°C for 40 s, and final extension at 72°C for 10 min. After the ligation step, barcoded samples belonging to each sub-library were pooled in a single tube and cleaned with microbeads. For size selection, 30 µl (3000 ng DNA) from each sublibrary + 10 µl loading dye were loaded into each sample well of the 2 % agarose gel cassette. The size selection step was performed in the Pippin prep (Sage Science) instrument with a fragment range of 300-500 bp. After running for 79 min, size-selected sub-library samples were obtained in the elution modules of the cassettes. After the pippin prep step, PCR was performed to enrich each sublibrary and to attach 5 indexes suitable for the Illumina platform to each sublibrary. In this study, we pooled 120 samples into 3 different genomic libraries (each containing 40 samples) to increase depth coverage in the sequencing process. The enriched and cleaned DNA libraries were sequenced by the Illumina NovaSeq 6000 platform to obtain raw paired-end reads (2 × 150 bp)

### ddRADseq data processing and variant calling

The *process_radtags* command embedded within the Stacks 2 program (Rochette et al., 2019) was employed with default parameters to assign each read to individuals. Demultiplexed reads were processed via the default settings of the fastp software (Chen et al., 2018) for adapter removal and quality trimming. Cleaned reads were aligned to the *Gallus gallus* reference assembly (GCA_016699485) via default parameters of the Bowtie2 algorithm (Langmead and Salzberg, 2012). BCFtools (Danecek et al., 2021) pipeline was utilized to call the variant in which only biallelic SNPs located on autosomal chromosomes with high read depth (20 ≤ D ≤ 500) and quality (Q ≥ 20) were retained. All insertions-deletions (InDels) and SNPs located on sex chromosomes and mitochondrial DNA as well as unplaced scaffolds were filtered from the dataset. The remaining SNPs were processed via the PLINK 1.9 program (Chang et al., 2015) in which SNPs with lower than 0.05 minor allele frequency (**MAF**) value and 90 % genotyping rate were filtered. Finally, individuals with a lower genotyping rate of 90 % regarding the remaining SNPs were filtered to obtain the final dataset.

### Genomic variability and population structure analyses

In this study, MAF, nucleotide diversity (**π**), observed heterozygosity (***H_O_***), expected heterozygosity (***H_E_***), and inbreeding coefficient (***F_IS_***) were calculated via VCFtools (Danecek et al., 2011) to assess the genomic variability in native Turkish chicken breeds and hybrid lines. SNeP v.1.1 software (Barbato et al., 2015) with default parameters was performed to evaluate historical changes in effective population size till 450 generations ago via the linkage disequilibrium approach. Principal component analysis (**PCA**) was carried out by the BITE package (Milanesi et al., 2017) while the results were plotted by the ggplot2 package (Wickham, 2011) for better visualization. A default pipeline of the SambaR package (De Jong et al., 2021) was used to calculate genetic distance values for drawing the Neighbour Joining (**NJ**) trees at population and individual levels. LEA package (Frichot and François, 2015) was run with different K values (from 2 to 5) to investigate population divergence via the ADMIXTURE algorithm in which the cross-entropy criterion (Alexander and Lange, 2011) was considered to detect the optimal number of ancestral populations. Similarly, population split and gene flow among studied chicken populations were assessed by TreeMix software (Pickrell and Pritchard, 2012) with the option of 0-5 migration events. TreeMix analysis was carried out with a block of 1,000 SNPs and 20 iterations per each migration event, while the optimal number of migrations was estimated by the OpTM package (Fitak, 2021). Of the aforementioned statistical packages, PLINK 1.9 (Chang et al., 2015), BITE (Milanesi et al., 2017), ggplot2 (Wickham, 2011), SambaR (De Jong et al., 2021), LEA (Frichot and François, 2015), and OpTM (Fitak, 2021) were run in the R environment (https://www.r-project.org).

## Results

### Sequenced data and SNP calling

The DNA libraries prepared according to ddRADseq were sequenced via Illumina NovaSeq 6000 instrument in which approximately 1.6 billion of 150 bp length short reads were recovered. The lowest (367 million) and highest (445 million) number of raw reads were detected in BROHIB and LAYHIB, respectively, while DNZ and GRZ had nearly similar values (Table 1). Approximately 5 % of the raw reads were removed after demultiplexing and quality trimming processes. A large part of clean reads was aligned to reference genome assembly in which the mean alignment rate was calculated as 93.51 % (Table 1). Coverage of depth values varied between 28.49x in BROHIB and 30.05x in the DNZ population with a mean of 29.45x.

**Table 1:** A summary of raw genetic data processing across the studied chicken populations.

Breed.	Raw reads	Clean Reads	Aligned reads	Alignment rate	Coverage of depth (x)
**DNZ**	394,279,640	376,614,522	353,151,437	93.77	30.05
**GRZ**	393,123,442	375,690,082	346,611,669	92.26	29.45
**BROHIB**	367,801,834	347,334,384	326,911,122	94.12	28.49
**LAYHIB**	445,115,974	419,576,493	393,898,411	93.88	29.82
**Mean**	400,080,223	379,803,870	355,143,160	93.51	29.45

The variant calling process confirmed that the number of common SNPs between Turkish chicken breeds and hybrid lines significantly differed. Indeed, a total of 5,643,455 SNPs and 50,078 InDels were detected across all samples while only 33,284 SNPs were filtered and common among both native Turkish and hybrid chickens. On the other hand, 94,208 SNPs were found to be common between DNZ and GRZ breeds. Therefore, in this study, two data sets namely 33 K (including 33,284 biallelic SNPs) and 94 K (including 94,208 biallelic SNPs across) were used for bioinformatics analyses. 33 K data set standed for 101 animals of DNZ (*n* = 29), GRZ (*n* = 29), BROHIB (*n* = 25), and LAYHIB (*n* = 18) populations genotyped in terms of 33,284 SNPs whereas 94 K data set included 94,208 SNPs across 58 samples of DNZ (*n* = 29)and GRZ (*n* = 29) breeds. The percentage of overlapped SNPs between two data sets was 100 % indicating that all SNPs created in 33 K data were also available in 94 K data.

### Genetic variability and trend in effective population size

The genetic variability parameters estimated for two different data sets were summarized in Table 2 in which MAF values ranged from 0.234 (BROHIB) to 0.375 (DNZ), while nucleotide diversity values were between 0.194 (BROHIB) and 0.305 (DNZ). Observed heterozygosity varied from 0.254 (BROHIB) to 0.386 (DNZ), whereas expected values ranged from 0.259 (BROHIB) to 0.375 (DNZ). Both data sets showed negative values for the inbreeding coefficient in DNZ and GRZ breeds, while this value was estimated at 0.118 and 0.241 for the LAYHIB and BROHIB populations, respectively (Table 2). Genetic diversity parameters in hybrid lines turned out to be lower than the values estimated for native Turkish chicken breeds. Both data sets (94 K and 33 K), on the other hand, confirmed that genetic variability in DNZ was higher than the values estimated in the GRZ breed (Table 2).

**Table 2:** Genetic variability parameters across the studied chicken populations.

Data set	Breed	MAF	π	*H_O_*	*H_E_*	*F_IS_*
**94K**	DNZ	0.335	0.305	0.350	0.362	−0.013
	GRZ	0.313	0.271	0.332	0.311	−0.050
**33K**	DNZ	0.375	0.278	0.386	0.375	−0.019
	GRZ	0.364	0.264	0.369	0.370	−0.011
	BROHIB	0.234	0.194	0.254	0.259	0.241
	LAYHIB	0.298	0.236	0.298	0.304	0.118

**Fig. 1:**
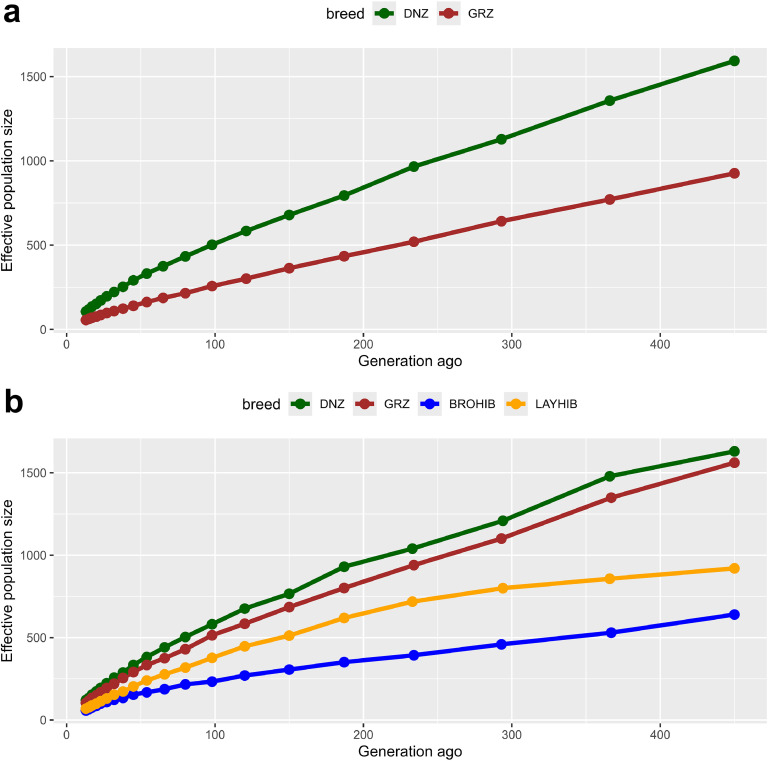
The distributions of historical effective population size per (a) 94 K and (b) 33 K SNP data. Abbreviations: DNZ, Denizli; GRZ, Gerze, BROHIB, broiler hybrid line; LAYHIB, layer hybrid line.

The distribution of effective population size until 450 generations ago was assessed via a linkage disequilibrium approach, in which the highest values were detected in the DNZ breed in all scenarios. According to data set 94 K, DNZ's effective population size was higher than 1,500 individuals, while GRZ turned out to be a descendant of a population whose effective size was lower than 1000 individuals (Fig. 1a). When the data set of 33 K was considered, the lowest effective population size was observed in BROHIB and LAYHIB for all generations, whereas this value was higher than 1,500 individuals for both DNZ and GRZ breeds (Fig. 1b).

### Population structure and divergence

**Fig. 2:**
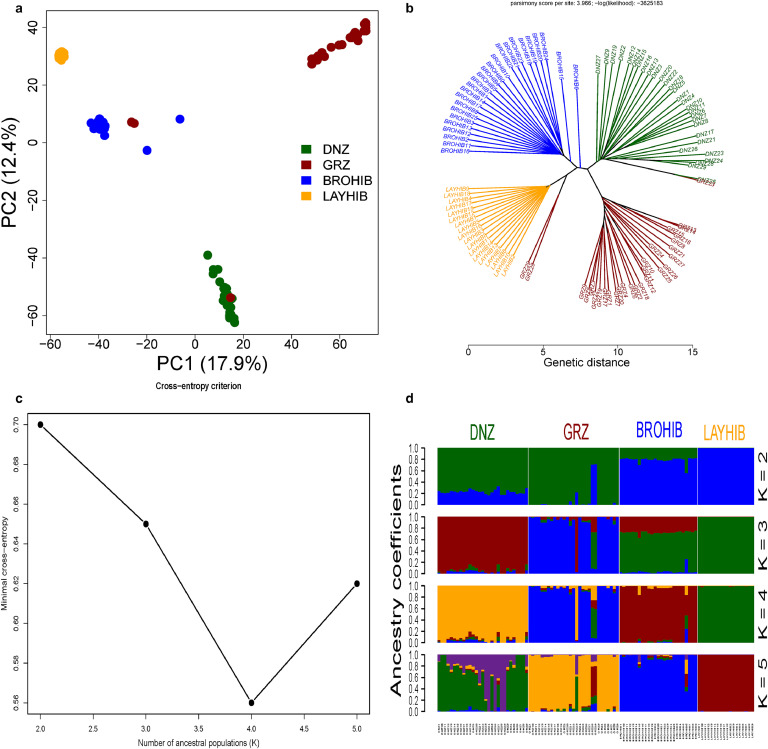
Analyses of population structure and divergence among the studied chicken population (a) PCA, (b) Nei’s genetic distance-based phylogenetic tree, (c) the distribution of cross-entropy criteria for different numbers of ancestral populations, and (d) ADMIXTURE analyses for different K values. Abbreviations: DNZ, Denizli; GRZ, Gerze, BROHIB, broiler hybrid line; LAYHIB, layer hybrid line.

The PCA analysis in which 17.9 % and 12.4 % proportion of total variation were attributed to components 1 and 2, respectively, was performed to identify the genetic relationship among native Turkish chicken breeds and commercial hybrid lines in which four distinctive clusters were detected (Fig. 2a). DNZ, LAYHIB, and BROHIB populations formed as nonoverlapping clusters while two and one samples of GRZ overlapped with commercial hybrids and DNZ, respectively. Similarly, the genetic distance-based phylogenetic tree has assigned individuals to four distinct clusters in which two and one samples of GRZ were found to be mixed with commercial hybrids and DNZ, respectively (Fig. 2b). Bayesian-based ADMIXTURE analysis was carried out for different K values ranging from 2 to 5 in which the optimal number of ancestral population was estimated for *K* = 4 according to the cross-entropy criterion (Fig. 2c). The cross-entropy criterion showed consistency with the findings of PCA and NJ tree analysis. At *K* = 4, all studied chicken populations seem to be descendants from four distinctive ancestral populations (Fig. 2d). It is noteworthy that native Turkish chicken breeds were clearly distinguished from commercial hybrid lines in all scenarios even when the ancestral number of the population was considered to be 2 (Fig. 2d).

**Fig. 3:**
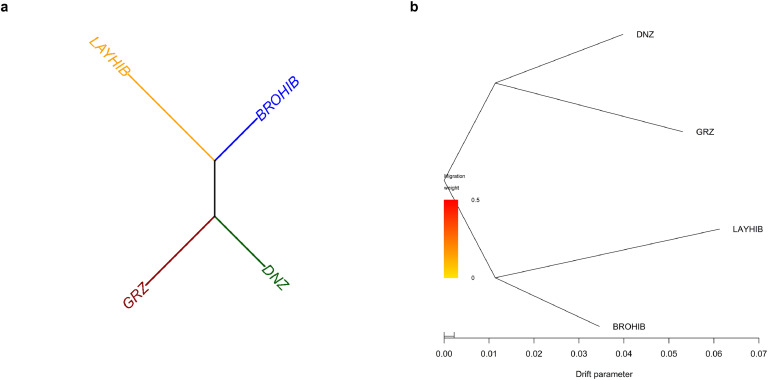
Phylogenetic tree analyses via (a) Nei’s genetic distance at breed level and (b) TreeMix-based maximum likelihood tree without migration events. Abbreviations: DNZ, Denizli; GRZ, Gerze, BROHIB, broiler hybrid line; LAYHIB, layer hybrid line.

In this study, phylogenetic trees per population were drawn based on both the genetic distance approach (Fig. 3a) and the TreeMix algorithm (Fig. 3b). Both approaches clearly separated native Turkish chicken breeds and commercial hybrid lines. Although migration between studied chicken populations up to 5 events was considered to be evaluated, no likelihood values were calculated for migration events from 1 to 5. This finding implies that no migration from one population to another was available.

## Discussion

Genetic diversity and population structure analysis in different local chicken breeds across the world have been mainly carried out with SNP array technologies (Cendron et al., 2021; Cho et al., 2023; Wu et al., 2024), while the current study investigated genomic variability and population structure in both native Turkish chicken breeds and commercial hybrid lines via ddRADseq technique for the first time. Therefore, the result of this study is of great importance for further studies because NGS platforms such as ddRADseq are a cost-efficient technique that allows parallel sequencing of millions of short reads belonging to hundreds of individuals. Indeed, Pértille et al. (2016) utilized NGS technologies to develop the CornellGBS protocol by which 67,096 bi-allelic SNPs with a 90 % call rate were recovered across 462 chickens. The authors highlighted that this protocol was not only cost-efficient (∼US$50/sample) but also a very powerful alternative to SNP array technology to improve genome-wide association and genomic selection studies in chickens. Similarly, in this study based on the ddRADseq technique, a total of 120 chickens (only 101 passed filtering process) were genotyped cost-efficiently, and the number of detected SNPs in native Turkish chicken breeds (94 K) showed consistency with previous studies carried out on different livestock species. Indeed, the number of SNPs ranging from 20,990 to 296,097 with high call rates were previously detected by the ddRADseq technique in chickens (Bateson et al., 2016), sheep (Karsli, 2024), goats (Simon et al., 2019), pigs (Li et al., 2020), cattle (Devadasan et al., 2020; Vineet et al., 2020; Demir et al., 2023), and buffalos (Tyagi et al., 2021). In this study, the number of detected SNPs significantly differs in native Turkish and commercial hybrid lines. Because a total of 33,284 SNPs were common between native Turkish and commercial hybrid lines whereas 60,924 SNPs were unique to local Turkish chickens. This finding not only indicates that local populations conserve a higher genetic variability across the genome compared to commercial chicken lines but also implies that NGS-based sequencing is an efficient way to screen breed-specific genomic variations, most of which are neglected in SNP array technologies.

In this study, genetic diversity analyses showed that native Turkish chicken breeds maintain greater genome-wide variability compared to commercial hybrid lines. Additionally, population structure analyses confirmed that these native breeds are genetically distinct from both one another and commercial hybrid lines. Similar findings were also reported in local chicken breeds in different countries. For example, a native Chinese chicken breed named Wenchang was reported to have higher values of genetic diversity parameters (π, *H_O_*, and *H_E_*) and to be genetically different from three commercial breeds (White Rock, White Leghorn, and White Plymouth Rock) via whole genome re-sequencing (Tian et al., 2023). Similar findings were also reported for the Bostwana (Machete et al., 2021) and Houdan (Liu et al., 2024) chicken breeds at the genome-wide level.

Several studies were available in the literature to clarify the genetic diversity and population structure in chicken breeds reared in Türkiye (Mercan and Okumuş, 2015; Karsli and Balcıoğlu, 2019; Bulut et al., 2023). For example, Kaya and Yıldız (2008) reported higher genetic diversity in the DNZ breed (*n* = 75) compared to GRZ (*n* = 50) chicken, while the NJ tree clarified that these breeds were genetically distinct based on 10 microsatellite markers. Özdemir et al. (2016) revealed a significant inbreeding coefficient in DNZ (0.214) and GRZ (0.143) breeds via 19 microsatellite loci, most likely due to the occurrence of nonrandom mating phenomena. The authors reported that native Turkish chicken breeds were of mainly higher genetic variability than native Italian breeds, while DNZ and GRZ were distinct clusters from both each other and native Italian chickens in population structure analysis (Özdemir et al., 2016). Similar findings were also reported in another study conducted by Özdemir and Cassandro (2018) via 19 microsatellite loci. A mtDNA-based study showed 24 polymorphic regions yielding 19 different haplotypes in native Turkish and Iranian chicken breeds in which only the E haplotype was detected in native Turkish chicken breeds (Meydan et al., 2016). The authors highlighted that native Turkish and Iranian chicken breeds originated from the same geographic zone. As seen, genetic diversity and population structure in DNZ and GRZ chicken breeds have been mainly evaluated by microsatellites and mtDNA diversity. However, as articulated by several studies (Bilginer et al., 2022; Demir et al., 2022), high-density SNP data representing the genome are required to reveal genetic variability and population structure in local populations sharing common past breeding practices. In this regard, this study is the first attempt to assess genetic variability and population structure in DNZ and GRZ chicken, the results of which are promising to reshape ongoing conservation programs. Indeed, some novel findings were detected via genome-wide ddRADseq data. For example, while the effects of nonrandom mating were reported in DNZ and GRZ via microsatellite markers (Özdemir et al., 2016), the current study proved that inbreeding coefficients were of negative values, indicating that mating occurs randomly. This contradiction may be explained by past breeding practices and/or the power of molecular genotyping methods. Indeed, ongoing conservation efforts may decrease inbreeding through comprehensive mating programs since 2016. Similarly, microsatellite markers may possess bias due to covering a small part of the genome to assess inbreeding in native Turkish chicken breeds, while NGS platforms allow to estimate accurately past breeding practices such as inbreeding level by monitoring the variations across all autosomes. In this study, comparatively higher genetic diversity and lower inbreeding were detected in DNZ and GRZ breeds than in commercial hybrid lines. This result is not surprising due to the fact that commercial hybrid lines have been subjected to selective breeding, leading to increased homozygosity across the genome. Therefore, the effective population size of commercial hybrid lines is expected to be lower compared to local chicken populations. The findings of the current study also supported this idea since the effective population size was higher than 1,500 individuals in DNZ and GRZ for 450-generation ago, while this value was lower than 1,000 individuals in commercial hybrid lines. The effective number of population size in the GRZ breed was lower than 1,000 individuals in 94 K data, while this value was higher than 1,500 individuals in 33 K data. It seems that the density of genetic data with different average MAF values may affect the results of linkage disequilibrium-based analysis. As reported by Kaya and Yıldız (2008), the current study also showed that DNZ conserves a higher genetic variability compared to GRZ breed. Previous studies showed that DNZ and GRZ were genetically different from each other and native Italian chicken breeds (Özdemir et al., 2016; Özdemir and Cassandro, 2018). Similarly, this study confirms that DNZ and GRZ are genetically distinct from each other and significantly differ from commercial hybrid lines. This finding supports the idea that local populations reared in certain geographic zones are exposed to diverse environmental conditions, triggering genetic mechanisms for the differentiation of the populations over generations.

In conclusion, conservation of the chicken populations with different genetic architectures is essential to sustain current production systems and guarantee meeting demand for animal-derived products in the future against diverse environmental challenges. This study has demonstrated that the genomic variability indicators in native Turkish chicken breeds were higher than those of commercial hybrid lines with lower inbreeding coefficients due to a most probably random mating process and the lack of selection studies. Both genetic variability and population structure analyses imply that ongoing conservation studies are promising to sustain local chicken breeding in Türkiye. However, special attention should be given to the GRZ breed whose genetic variability parameters were slightly lower than the DNZ breed. Moreover, these parameters as well as inbreeding levels should be periodically screened via accurate molecular genotyping methods to take necessary precautions against future challenges. This study also proves that the ddRADseq technique offers accurate results regarding genetic diversity and population structure analysis in a cost-efficient manner which seems to become one of the most widespread molecular genotyping methods in different livestock species in the future.

## Data availability

The dataset used in this study is available upon request via Material Transfer Agreement (MTA) signed by the corresponding author for only scientific purposes.

## Declaration of competing interest

The authors declare no potential conflict of interest.
